# Can we identify which patients are likely to have septic arthritis with borderline synovial fluid cell counts?

**DOI:** 10.5194/jbji-11-287-2026

**Published:** 2026-05-19

**Authors:** Boshen Liu, Michael Raffetto, Eric J. Abbenhaus, Gavin S. Hautala, William R. Taylor, Lucy Bowers, Paul Edward Matuszewski

**Affiliations:** 1 Department of Orthopaedic Surgery, University of Kentucky, 740 S Limestone St, K423, Lexington, KY 40508, USA

## Abstract

**Objective**: Septic arthritis (SA) is a clinical emergency that requires prompt diagnosis and surgical treatment to minimize morbidity and mortality. A synovial fluid cell count (SFCC) greater than 50 000 mm^−3^ has been a threshold used to diagnose SA. However, patients with lower cell counts may have culture-positive septic arthritis, resulting in missed or delayed diagnoses. The purpose of this study was to assess the risk of culture-positive SA in patients with synovial fluid analyses that would typically be considered inconclusive and determine what factors may increase the risk of SA. **Methods**: Patients with SFCC < 50 000 mm^−3^ were retrospectively assessed at a single academic institution between 2010 and 2019. Laboratory measures such as white blood cell count (WBC), C-reactive protein (CRP), erythrocyte sedimentation rate (ESR), SFCC polymorphonuclear leukocyte percent (PMN), and patient factors and comorbidities were evaluated to determine a threshold for diagnosing SA in this patient population. **Results**: A total of 199 of 849 patients met inclusion criteria. There were 31 (15.6 %) cases of SA. SFCC and PMN thresholds of 20 000 cell mm^−3^ and 70 %, respectively, maximized sensitivity and specificity of SA detection. Prior history of SA, inflammatory arthritis and/or crystalline arthropathy was associated with increased risk of SA. **Conclusion**: The traditional SFCC threshold of 50 000 cells mm^−3^ may not be a reliable diagnostic criterion for all patients. Prior history of SA, inflammatory arthritis, or crystalline arthropathy is associated with increased risks of SA. It may be more appropriate to have lower diagnostic thresholds to clinically diagnose SA in these subsets of patient populations.

## 1 Introduction

Septic arthritis (SA) is a clinical emergency with significant morbidity and mortality. In 2012, SA was responsible for 16 382 emergency room (ER) visits, resulting in significant financial and healthcare burden (Singh and Yu, 2018). The most common cause of SA arises from hematogenous seeding (Morgan et al., 1996). The inflammatory response from SA, with subsequent release of proteolytic enzymes, can lead to irreversible cartilage and subchondral bone loss in as little as 3 d, if not properly diagnosed and treated (Shirtliff and Mader, 2002). In addition, case fatality rate associated with SA can be as high as 11 % (Mathews et al., 2010). Therefore, a prompt diagnosis, initiation of antibiotics, and surgical debridement and irrigation should be performed as soon as possible to decrease morbidity and mortality.

Historically, the diagnosis of SA has been made using a combination of clinical history and presentation, physical examination, laboratory data, and arthrocentesis, with arthrocentesis being the most objective criteria and the gold standard for definitive diagnosis. A synovial fluid cell count (SFCC) greater than 50 000 cells mm^−3^ is accepted to be diagnostic of SA in clinical practice. However, the evidence supporting 50 000 cells mm^−3^ as the threshold for SA is limited. It has been reported that synovial fluid findings can be highly variable, and there can be significant overlap in patients with the underlying diagnosis of gout, pseudogout, or rheumatoid arthritis (Yu et al., 2003; Lenski and Scherer, 2014). Conversely, septic arthritis can present with SFCC < 50 000 cells mm^−3^ in patients who are unable to mount an adequate leukocytic response – such as immunocompromised patients. Coutlakis et al. (2002) reported that rates of SA with SFCC < 50 000 mm^−3^ can be as high as 5 %. It is unclear whether a lowered threshold should be used for the aforementioned subsets of patients in order to avoid a missed or delayed diagnosis of SA.

The primary objective of this research study was to assess the risk of culture-positive SA in patients with synovial fluid analyses that would typically be considered inconclusive and to identify which patient-specific factors may modify the risk of SA. Furthermore, our secondary objective was to determine a threshold of SFCC and PMNs that can be utilized in making an accurate diagnosis of SA in at-risk patient populations.

## 2 Methods

### 2.1 Study design and patient population

After Institutional Review Board (IRB) approval, a retrospective study was performed at a single academic institution and included all patients who underwent joint arthrocentesis between July 2010 and December 2019. A total of 849 patients were identified. The patients were then screened to exclude those with active septic arthritis, patients who did not undergo an aspiration, patients with SA involving a prosthetic total joint replacement, patients with therapeutic joint aspirations, and patients with SA with SFCC greater than 50 000 cells mm^−3^. A total of 199 patients met the criteria and were used for this analysis.

For the patients who met inclusion criteria, demographic data (age, sex, BMI, tobacco use), medical history (HIV, diabetes mellitus, liver disease, chronic kidney disease, history of SA, osteomyelitis or cellulitis, inflammatory arthritis, immunocompromised status), social history (self-reported oral antibiotics use within 2 weeks of presentation, history of intravenous drug use, presence of hardware from prior trauma), laboratory measures (white blood cell count (WBC), C-reactive protein (CRP), erythrocyte sedimentation rate (ESR)) and synovial fluid aspiration results (synovial fluid nuclear cell counts (SFCCs), percent polymorphonuclear leukocytes (PMNs), cultures, crystals, gram stain) were recorded. Laboratory data were analyzed within 1 h of obtaining the synovial samples by our institution's microbiology laboratory.

### 2.2 Septic arthritis

The diagnosis of SA was determined based on positive cultures from initial joint arthrocentesis or cultures obtained during a surgical debridement and irrigation. Patients with frank purulence upon aspiration or surgical approach or features consistent with septic arthritis (e.g., draining sinus) were also regarded as having a diagnosis of septic arthritis.

### 2.3 Statistical analysis

Patients were categorized according to quartiles of SFCC at clinical examination. Continuous variables were summarized as mean ± standard deviation or median (percentile_25–75_), and categorical variables are presented as absolute numbers ( n ) and percentages. Clinical characteristics were compared across these four groups using ANOVA or Kruskall–Wallis tests as appropriate. A restricted cubic spline function with three knots was used to examine the potential non-linear dose-response association of SFCC with SA. To further evaluate the power of SFCC to predict SA, eight arbitrary values of SFCC (10 000, 15 000, 20 000, 25 000, 30 000, 35 000, 40 000, 45 000 cells mm^−3^) and seven arbitrary values of PMNs from 30 % to 90 % were selected to study their associations with SA using a multinomial logistic model. The area under the curve for receiver operating characteristic (ROC) and a 95 % confidence interval were calculated for sensitivity and specificity at different arbitrary thresholds for SFCC and PMNs. In the adjusted models, the associations were further corrected for age, sex, BMI, smoking status, diabetes, chronic kidney disease, liver disease, history of septic arthritis, history of inflammatory arthritis, immunocompromised state, IV drug use, CRP and ESR. In addition, the variables strongly associated with nucleated cell count were assessed using a stepwise forward multinomial logistic model. All statistical analyses were performed using STATA^®^ Statistical Software version 16.0 (STATA Corp, College Station, Texas).

## 3 Results

### 3.1 Patient demographics

Overall, 199 patients who underwent joint aspiration and met the inclusion criteria were included in the final analysis. The average age was 51 ± 17, and the majority of patients were male (125, 63 %). The average BMI was 29.3 ± 8.2, and 12 % of patients reported current tobacco use. Average Charlson comorbidity index (CCI) was 2.7 ± 2.7, and median hospital length of stay was 0.8 d [0.3, 8.2]. The distribution of aspirated joints by anatomic location was as follows: knee (151 aspirates, 76 %); hip (36 aspirates, 18 %); and other joints, including shoulder, ankle and elbow (12 aspirates, 6 %). Patient demographics were categorized according to synovial fluid cell count quartiles and are summarized in Table A1 in the Appendix.

### 3.2 Coexistent conditions

Table A2 lists a number of coexistent conditions which were frequently identified. In our cohort, 49 (25 %) patients had crystalline arthropathy, 13 (7 %) had inflammatory arthritis and 16 (8 %) had purulent aspirate upon arthrocentesis. Higher levels (highest quartile) of nucleated cell counts were associated with higher prevalence of joint purulence, intra-articular crystals and inflammatory arthritis. Additionally, patients with increasing SFCC levels had longer hospital stays and were more likely to have a prior history of SA. A total of 72 (36 %) patients reported recent use of antibiotic medications for the treatment of pathologies other than septic arthritis.

### 3.3 Laboratory testing

**Figure A1:**
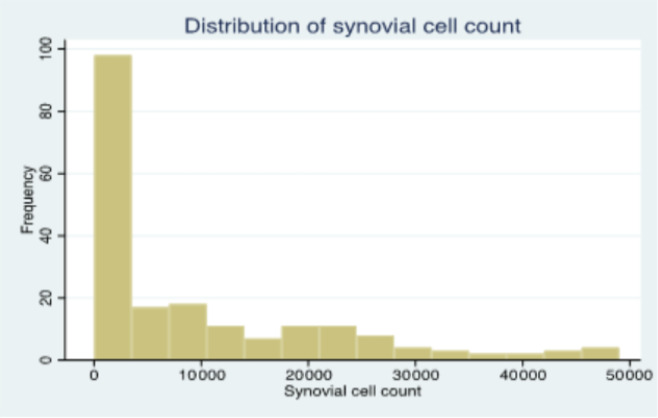
Distribution of synovial fluid cell counts (cells mm^−3^) among patients.

Overall, the median SFCC was 4345 cells mm^−3^ (range: 356–46 942), which is further demonstrated in Fig. A1 in the Appendix, indicating that the distribution of nucleated cell counts was highly skewed. Aspiration volumes did not change across quartiles of nucleated cell count. Patients with increased SFCC were more likely to have higher levels of CRP, ESR and aspirate PMN percentage. Only 7 of the 31 patients with SA (22.6 %) had a positive synovial fluid gram stain immediately following joint aspiration. The most common infectious species was *Staphylococcus aureus* (41.9 %). Only one case was diagnosed off of visualized aspirate purulence alone. In that case, more purulence was encountered in the operating room without a clear sign of synovitis. A total of 22 cases were diagnosed off of aspirate culture alone (no definitive purulence on aspiration). Of these 22 cases, 15 had findings in the operating room that were consistent with septic arthritis (infected/necrotic synovium, chondrolysis, etc.).

**Figure A2:**
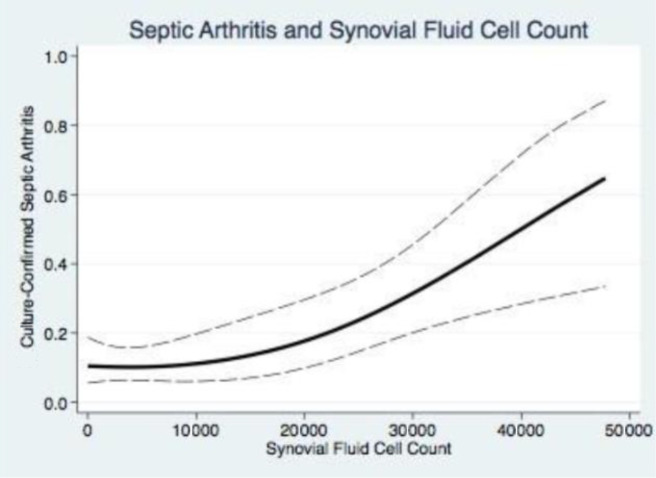
Relationship of synovial fluid cell counts with septic arthritis. Cubic spline analysis demonstrating increasing probability of septic arthritis with increasing SFCC values. The continuous line demonstrates the non-linear relationship of SFCC and SA. The dashed lines indicate the upper and lower limits of the spline model.

**Figure A3:**
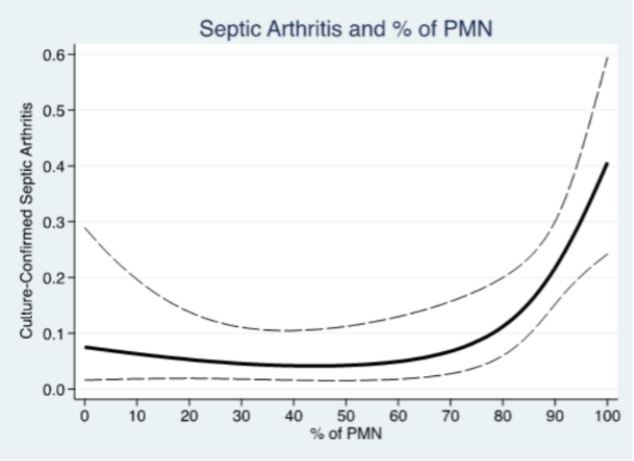
Relationship of % of PMN with septic arthritis. Cubic spline analysis demonstrating increasing probability of septic arthritis with increasing % PMN values. The continuous line demonstrates the non-linear relationship of % PMN and SA. The dashed lines indicate the upper and lower limits of the spline model.

Of the 199 subjects included, there were 31 (16 %) cases of SA. Patients with a higher level of SFCC were more likely to have SA. Figures A2 and A3 display the restricted cubic spline plot that graphically describes the independent exponential association between SFCC and PMNs with SA. Furthermore, eight arbitrary threshold values of nucleated cell count (from 10 000 to 50 000) and seven arbitrary thresholds for PMNs (from 30 % to 100 %) were studied in the association analysis (Table A3). SFCC of 15 000 mm^−3^ and 50 % PMN were the lowest arbitrary threshold values to have a statistically significant association with SA in the univariate model, (odds: 2.39, CL: 1.08–5.31, p=0.03 and odds: 3.68, CL: 1.06–12.71, p=0.04 , respectively). The likelihood ratio increased with increases in values of nucleated cell count and PMNs as well, which remained statistically significant throughout all threshold values. After adjusting for covariates, including age, sex, BMI, smoker, Charlson comorbidity index, history of diabetes, chronic kidney disease, liver disease and septic arthritis, inflammatory arthritis, immunocompromised, IV drug use, HIV, and hepatitis C, as well as laboratory tests such as WBC, CRP and ESR, SFCC of 20 000 mm^−3^ and 60 % PMNs were the lowest arbitrary threshold values to have a statistically significant association with SA (odds: 4.55, CL: 1.18–17.47, p=0.03 and odds: 8.66, CL: 1.22–61.53, p=0.03 , respectively). Similarly, the diagnostic performance of SFCC and PMNs, including sensitivity, specificity and area under the curve (AUC) from the ROC analysis, was consistent with the results that we observed in the association analysis (Table A5). The maximized combinations (AUC) of sensitivity and specificity on the ROC curve were 0.62 (CI 0.52–0.71 for SFCC at the threshold of 20 000 mm^−3^) and 0.66 (CI 0.59–0.72 for PMN at the threshold of 70 %). Through a stepwise selection procedure, a history of septic arthritis, inflammatory arthritis and crystalline arthropathy was found to be associated with elevation of nucleated cell count and subsequent increased risk of SA. An increase in PMNs in SA was more likely associated with the history of SA, crystalline arthropathy and blood tests of CRP and ESR (Table A4).

## 4 Discussion

Septic arthritis continues to be associated with significant morbidity and mortality even with effective treatment (Ferrand et al., 2016). Although uncommon, the clinical presentation of SA patients can mimic other benign joint pathology such as crystalline arthropathy, inflammatory arthritis exacerbation, or other rare causes such as local aspiration or prior corticosteroid injection (Hunter and Blyth, 1999). The task of clinicians is to properly diagnose and subsequently treat patients for which there is a clinical suspicion of SA. This can be made challenging when the history and presentation are suggestive of SA but joint aspirate analysis is inconclusive or culture data are not available in a timely fashion. A delayed diagnosis may lead to irreversible joint damage, while over-diagnosis may cause patients to undergo unnecessary medical and surgical treatments. Therefore, accurate diagnosis is crucial. In clinical practice, a positive culture from the affected joint serves as the gold standard diagnostic test. However, this may take days to produce a final result. In order to initiate prompt and appropriate treatment, alternative laboratory data are often utilized to make a clinical diagnosis. In addition to analysis of systemic inflammatory markers such as CRP, ESR and WBC, the SFCC and percentage of PMNs from arthrocentesis are frequently used to arrive at the clinical diagnosis of septic arthritis in the absence of a positive aspirate culture. Historically, SFCC above 50 000 cells mm^−3^ with greater than 75 % PMNs raised concern for SA in a native joint (Jokic et al., 2020; Long et al., 2018). However, the literature to support this threshold is rather inconclusive. In contrast, retrospective studies have reported that only 32.5 % of patients with culture-positive SA had a nucleated cell count above 50 000 mm^−3^ (Rasmussen et al., 2020). Furthermore, Coutlakis et al. (2002) reported SA with nucleated cell counts lower than 50 000 cells mm^−3^ can be as high as 5 %. Therefore, the diagnosis of SA using an SFCC threshold of 50 000 mm^−3^ may be more dogmatic than evidence based. The purpose of our study was to retrospectively review patients who underwent joint aspirations with SFCC less than 50 000 mm^−3^ and determine if this value is a valid threshold in clinical practice.

In our association analysis of 199 joint aspirations, we found that values of SFCC greater than 15 000 mm^−3^ (OR: 2.39 [1.08–5.31]; P<0.03 ) and PMNs greater than 50 % (OR: 3.68 [1.06–12.71]; p<0.04 ) were associated with an increased risk of having SA without any adjustment. The threshold value of SFCC and PMNs became 20 000 mm^−3^ (OR: 4.55 [1.18–17.47]; p<0.03 ) and 60 % (OR 8.66 [1.22–61.53]; p<0.03 ), respectively, after adjusting for important clinical correlates (Table A3). This contrasts with a prior meta-analysis by Carpenter et al. (2011); they reported an SFCC of 50 000–100 000 cells mm^−3^ has 64 % probability of developing SA, while SFCC > 100 000 mm^−3^ significantly elevated that probability to 83 %. Our study results have not only supported the importance of SFCC and PMNs in clinical diagnosis of SA, but also defined a lower threshold value for SFCC and PMNs in our culture-confirmed SA patient cohort. These results may provide valuable insight for properly diagnosing at-risk patients with SA and allow for prompt treatment.

Several studies (Rasmussen et al, 2020; Carpenter et al., 2011; Kinugasa et al., 2020) have evaluated the diagnostic power of nucleated cell counts in SA patients but have reported controversial results. Margaretten et al. (2007) evaluated 14 studies of laboratory testing for diagnosing SA and reported that the aspirate SFCC and PMN measures were the most useful diagnostic markers for SA. With an increase in the nucleated cell count from 25 000 to greater than 50 000 mm^−3^, the likelihood ratio of SA increased from 2.9 to 7.7. On the same synovial fluid samples, the likelihood ratio of SA with PMN values less than 90 % was 0.34 but reached 3.4 with PMN values greater than 90 %. Carpenter et al. (2011) have also reported a lower likelihood ratio of 1.06 if the nucleated cell count was 25 000–50 000 cells mm^−3^, rising to 3.59 when the nucleated cell count was above 50 000 cells mm^−3^. Li et al. (2007) reviewed 156 patients who underwent arthrocentesis and included only those with positive cultures or intraoperative findings of infection. SFCC of 50 000 cells mm^−3^ from their study did not provide an accurate diagnosis of SA, with a sensitivity of only 50 %, while a lower threshold value of 17 500 mm^−3^ had maximized the sensitivity (83 %) and specificity (67 %). Results from our study demonstrate similar trends. Our study has highlighted that the likelihood of SA significantly increases at a lower SFCC threshold of 20 000 mm^−3^ after adjusting for medical comorbidities (OR: 4.55, CI: 1.18–17.47; p<0.03 ). In addition, our study has also shown that PMNs greater than 60 % serve as an even stronger predictor of SA (OR: 8.66, CI: 1.22–61.53; p<0.03 ). The diagnostic performances of AUC for SFCC and PMNs that maximized the combination of sensitivity and specificity were the highest at a threshold of 20 000 mm^−3^ for SFCC and 70 % for PMNs (Table A5), which were consistent with the results that we observed in the association analysis (Table A3). We acknowledge that values of the ROC were somewhat low. This study focuses on a pathology that is not always straightforward to diagnose. As such, interpretation of multiple clinical and laboratory findings is often used to reach a conclusion. In light of this, we think that 82 % specificity and an adjusted odds ratio of 4.55 are relatively high for a proposed SFCC cutoff of 20 000 cells. Although increasing the cutoff values for SFCC and PMN thresholds could reduce the number of false positives, it could also increase the number of missed and/or delayed diagnosis. Based on our results, we would suggest lowering the diagnostic threshold of 20 000 mm^−3^ for SFCC and 70 % for PMNs as these cutoffs were correlated with significantly increased likelihood of SA in the population studied.

Numerous risk factors have been reported to be associated with increases in SFCC and/or PMNs in SA patients. They include age, recent joint surgery, intra-articular corticosteroid injection, inflammatory arthritis and immunocompromised states (including chronic kidney disease, liver diseases and HIV) (Kaandorp et al., 1995; Zeller et al., 2007). For example, Edwards et al. (2007) suggested that the incidence of SA is 12.9 times higher in patients with inflammatory arthritis than in those without. Kennedy et al. (2015) found that patients with rheumatoid arthritis develop SA at a rate 4–8 times that of those without RA. Interestingly, 23.4 % of their cohort had SA with underlying crystalline arthropathy. Our study has identified similar risk factors that were associated with changes in SFCC or PMNs, highlighting the importance of identifying at-risk patients when SA is clinically suspected.

There were some limitations with this study. It had inherent weaknesses generally expected with a retrospective study design. Given that it was not a prospective study, a power analysis was not performed. Multiple joints were included in the study, which may have different pathological responses to infection. Information regarding the degree of immunocompromised status, the current state of immunocompromising medication administration, steroid use and severity of diabetes was not recorded. In our study, 47/199 (23.6 %) had a history of prior intra-articular steroid injection. A total of 14/31 (45 %) patients with SA in this study had a prior history of steroid injection. However, information regarding injection location, dosing frequency or time to last injection was not recorded. In addition, we were limited in the resolution of our findings due to the number of cases fitting our inclusion criteria, although this was one of the largest studies to date. This limited our ability to comment on some of our findings with statistical confidence, such as the potential ability of laboratory testing to identify SA, as evidenced by the wide 95 % confidence intervals associated with the likelihood ratios in Table A3. However, previous studies have also included multiple joints and did not have a large sample size (Ferrand et al., 2016; Carpenter et al., 2011; Kinugasa et al., 2020; Margaretten et al., 2007; Li et al., 2007; Gupta et al., 2003). In prior studies, both crystalline and inflammatory arthropathies were found to be implicated in the diagnosis of SA. In our study, nearly 25 % of patients had a history of crystalline arthropathy. While our data analysis included inflammatory arthropathy as a covariate in our regression, a history of crystalline arthropathy was not included as a covariate. We also acknowledge that in our study, SFCC was skewed towards lower values. While this likely helps with evaluation of lower SFCC and PMN values, it may limit the interpretation of higher values. Despite these limitations, given the signal that was found, our findings offer important diagnostic insight as they prompt clinicians to consider a lower threshold of SFCC and PMNs when making the diagnosis of SA, especially in at-risk patient populations. A future prospective study may better elucidate this.

## 5 Conclusion

In conclusion, both SFCC and PMNs are important laboratory tests in diagnosing SA. A lowered threshold value of SFCC (20 000 mm^−3^) and PMNs (60 %) may be more appropriate for patients at risk. Heightened clinical suspicion should be raised for patients with underlying inflammatory arthritis, a history of crystalline arthropathy and/or a history of septic arthritis. Notably, the culture-positive SA patients in this study were selected based on their nucleated cell count under 50 000 mm^−3^. Clinical evaluation, such as patient history, physical examination and laboratory values, is recommended for early diagnosis and treatment of SA.

## Data Availability

A publicly available database/registry was not used.

## Appendix A

**Table A1:** Basic patient demographics categorized by quartile of synovial fluid cell counts (SFCCs). Bold text indicates statistically significant p value ( <0.05 ).

	Overall (SFCC) 4345 [356, 16 942] ( n=199 )	Quartile 1 (SFCC) 76 [44, 180] ( n=50 )	Quartile 2 (SFCC) 933 [639, 1762] ( n=50 )	Quartile 3 (SFCC) 8232 [6286, 11 610] ( n=50 )	Quartile 4 (SFCC) 25 630 [22 000, 32 750] ( n=49 )	p for trend
Demographic						
Age (mean ± SD)	51 ± 17	48 ± 17	50 ± 15	52 ± 19	51 ± 17	0.27
Sex, male ( n , %)	125 (62.8 %)	33 (66.0 %)	27 (54.0 %)	36 (72.0 %)	29 (59.2 %)	0.94
Race ( n , %)						0.42
White	168 (84.4 %)	40 (80.0 %)	43 (86.0 %)	43 (86.0 %)	42 (85.7 %)	
African American	28 (14.1 %)	9 (18.0 %)	6 (12.0 %)	7 (14.0 %)	6 (12.2 %)	
Asian	1 (0.5 %)	1 (2.0 %)	0 (0.0 %)	0 (0.0 %)	0 (0.0 %)	
Middle Eastern	1 (0.5 %)	0 (0.0 %)	1 (2.0 %)	0 (0.0 %)	0 (0.0 %)	
Unreported	1 (0.5 %)	0 (0.0 %)	0 (0.0 %)	0 (0.0 %)	1 (2.0 %)	
BMI	29.3 ± 8.2	30.5 ± 11.5	31.0 ± 6.8	27.7 ± 6.8	27.9 ± 6.2	**0.034**
Weight	87.6 ± 28.0	92.6 ± 40.2	92.0 ± 23.5	82.9 ± 23.1	82.9 ± 19.9	**0.032**
Height	172.4 ± 10.7	173.0 ± 9.9	172.2 ± 11.7	172.3 ± 10.9	172.2 ± 10.8	0.72
Tobacco use ( n , %)						0.81
Never	104 (53.1 %)	27 (54.0 %)	23 (46.9 %)	29 (60.4 %)	25 (51.0 %)	
Former	68 (34.7 %)	19 (38.0 %)	20 (40.8 %)	13 (27.1 %)	16 (32.7 %)	
Current	24 (12.2 %)	4 (8.0 %)	6 (12.2 %)	6 (12.5 %)	8 (16.3 %)	
Charlson comorbidity index	2.7 ± 2.7	2.8 ± 2.8	2.9 ± 3.0	2.5 ± 2.4	2.6 ± 2.7	0.60
Length of hospital stay	0.8 [0.3, 8.2]	0.5 [0.2, 4.0]	0.8 [0.4, 9.9]	0.7 [0.3, 6.1]	4.0 [0.4, 12.5]	**0.01**
Affected joint						0.44
Knee	151 (75.9 %)	35 (70.0 %)	38 (76.0 %)	42 (84.0 %)	36 (73.5 %)	
Hip	36 (18.1 %)	10 (20.0 %)	9 (18.0 %)	7 (14.0 %)	10 (20.4 %)	
Other (shoulder,elbow, ankle)	12 (6.0 %)	5 (10.0 %)	3 (6.0 %)	1 (2.0 %)	3 (6.1 %)	

Basic demographic data of subjects grouped by SFCC quartile. Data are presented as means ± SD or n (%) and median (25th and 75th percentiles) unless indicated otherwise.

**Table A2:** Clinical features, laboratory measures and cultured species categorized by quartile of synovial fluid cell counts (SFCCs). Bold text indicates statistically significant p value ( <0.05 ).

	Overall 4345 [356, 16 942] ( n=199 )	Quartile 1 76 [44, 180] ( n=50 )	Quartile 2 933 [639, 1762] ( n=50 )	Quartile 3 8232 [6286, 11 610] ( n=50 )	Quartile 4 25 630 [22 000, 32 750] ( n=49 )	p for trend
***Clinical features***						
**SA**	**31 (15.6 %)**	**3 (6.0 %)**	**6 (12.0 %)**	**9 (18.0 %)**	**13 (26.5 %)**	<0.001
**Purulence on initial aspiration**	**16 (8.0 %)**	**1 (2.0 %)**	**2 (4.0 %)**	**3 (6.0 %)**	**10 (20.4 %)**	<0.001
Recent antibiotic use	72 (36.2 %)	21 (42.0 %)	17 (34.0 %)	15 (30.0 %)	19 (38.8 %)	0.65
**Crystal arthropathy**	**49 (24.6 %)**	**5 (10.0 %)**	**9 (18.0 %)**	**20 (40.0 %)**	**15 (30.6 %)**	**0.002**
Presence of hardware	18 (9.0 %)	5 (10.0 %)	5 (10.0 %)	5 (10.0 %)	3 (6.1 %)	0.53
**Inflammatory arthritis**	**13 (6.5 %)**	**0 (0.0 %)**	**2 (4.0 %)**	**4 (8.0 %)**	**7 (14.3 %)**	**0.003**
Immunocompromised	42 (21.1 %)	12 (24.0 %)	12 (24.0 %)	8 (16.0 %)	10 (20.4 %)	0.47
IV drug user	25 (12.8 %)	7 (14.0 %)	4 (8.2 %)	6 (12.5 %)	8 (16.3 %)	0.60
Diabetes	29 (14.6 %)	6 (12.0 %)	8 (16.0 %)	5 (10.0 %)	10 (20.4 %)	0.40
Chronic kidney disease	24 (12.1 %)	5 (10.0 %)	10 (20.0 %)	3 (6.0 %)	6 (12.2 %)	0.72
**HIV**	**6 (7.4 %)**	**4 (16.0 %)**	**1 (5.9 %)**	**1 (5.3 %)**	**0 (0.0 %)**	**0.045**
Liver diseases	11 (5.5 %)	2 (4.0 %)	3 (6.0 %)	3 (6.0 %)	4 (8.2 %)	0.41
Osteomyelitis	19 (9.5 %)	5 (10.0 %)	4 (8.0 %)	4 (8.0 %)	6 (12.2 %)	0.72
Cellulitis	14 (7.0 %)	3 (6.0 %)	5 (10.0 %)	3 (6.0 %)	3 (6.1 %)	0.83
**History of septic arthritis**	**30 (15.1 %)**	**5 (10.0 %)**	**6 (12.0 %)**	**7 (14.0 %)**	**12 (24.5 %)**	**0.047**
***Laboratory***						
WBC	12.6 ± 8.3	11.5 ± 9.4	11.7 ± 6.0	12.0 ± 5.5	14.7 ± 10.6	0.09
**CRP**	9.0±9.4	7.1±9.9	4.5±5.4	11.3±9.9	12.1±9.6	**0.002**
**ESR**	54.6±32.9	44.9±31.1	50.3±32.7	57.7±31.4	64.4±34.1	**0.009**
Aspiration vol	27.4 ± 33.9	20.5 ± 45.9	24.1 ± 26.7	36.3 ± 28.6	28.9 ± 32.2	0.13
**% PMNs**	67±32	42±33	54±33	81±18	91±8	<0.001
**Gram stain**	**7 (3.6 %)**	**1 (2.1 %)**	**0 (0.0 %)**	**0 (0.0 %)**	**6 (12.2 %)**	**0.01**
***Septic arthritis cases***						
*Enterococcus faecalis*	1 (3.2 %)	0 (0.0 %)	0 (0.0 %)	0 (0.0 %)	1 (8.3 %)	
Gram-positive rods	1 (3.2 %)	1 (25 %)	0 (0.0 %)	0 (0.0 %)	0 (0.0 %)	
*Kocuria rhizophola*	1 (3.2 %)	0 (0.0 %)	1 (14.3 %)	0 (0.0 %)	0 (0.0 %)	
MRSA	5 (16.1 %)	1 (25 %)	1 (14.3 %)	1 (12.5 %)	2 (16.7 %)	
MSSA	8 (25.8 %)	0 (0.0 %)	1 (14.3 %)	2 (25 %)	5 (41.2 %)	

**Table A2:** Continued.

	Overall 4345 [356, 16 942] ( n=199 )	Quartile 1 76 [44, 180] ( n=50 )	Quartile 2 933 [639, 1762] ( n=50 )	Quartile 3 8232 [6286, 11 610] ( n=50 )	Quartile 4 25 630 [22 000, 32 750] ( n=49 )	p for trend
***Septic arthritis cases***						
Other staphylococcus species	8 (25.8 %)	1 (25 %)	2 (28.6 %)	4 (50 %)	1 (8.3 %)	
Purulent aspirate with negative culture	4 (12.9 %)	0 (0.0 %)	0 (0.0 %)	1 (12.5 %)	3 (25 %)	
*Serratia mascerans*	1 (3.2 %)	0 (0.0 %)	1 (14.3 %)	0 (0.0 %)	0 (0.0 %)	
Streptococcal species	2 (6.5 %)	1 (25 %)	1 (14.3 %)	0 (0.0 %)	0 (0.0 %)	

Comparison of clinical features, laboratory measures and cultured species using ANOVA and Kruskall–Wallis tests, categorized by quartile of SFCC. Data are presented as means ± SD or n (%) and median (25th and 75th percentiles) unless indicated otherwise.

**Table A3:** Association of septic arthritis with synovial fluid cell counts at different thresholds. Bold text indicates statistically significant p value ( <0.05 ).

SFCC	Unadjusted (OR, 95 % CI)	p	|Z|	Adjusted^*^ (OR, 95 % CI)	p	|Z|
> 10 000	2.09 (0.96–4.55)	0.06	1.86	1.90 (0.52–6.96)	0.33	0.98
> 15 000	2.39 (1.08–5.31)	0.03	2.14	2.42 (0.68–8.63)	0.17	1.36
>20000	**3.32 (1.47–7.51)**	**0.004**	**2.89**	**4.55 (1.18–17.47)**	**0.03**	**2.21**
>25000	**4.86 (1.93–12.20)**	**0.001**	**3.36**	**9.88 (1.93–50.47)**	**0.006**	**2.75**
>30000	**6.96 (2.38–20.34)**	<0.001	**3.54**	**7.23 (1.29–40.48)**	**0.02**	**2.25**
>35000	**11.96 (3.26–43.92)**	<0.001	**3.74**	**18.81 (1.90–186.72)**	**0.01**	**2.51**
>40000	**24.21 (4.75–123.40)**	<0.001	**3.83**	**20.63 (1.96–217.51)**	**0.01**	**2.52**
>45000	**17.89 (1.80–178.16)**	**0.01**	**2.46**	**33.49 (1.45–772.52)**	**0.03**	**2.19**
% PMN	Unadjusted (OR, 95 % CI)	p	|Z|	Adjusted^*^ (OR, 95 % CI)	p	|Z|
>30 %	2.76 (0.79–9.60)	0.11	1.59	1.87 (0.26–13.56)	0.53	0.62
>40 %	3.15 (0.91–10.92)	0.07	1.81	2.38 (0.33–17.22)	0.39	0.86
> 50 %	3.68 (1.06–12.71)	0.04	2.06	2.88 (0.42–19.66)	0.28	1.08
>60%	**4.63 (1.34–15.93)**	**0.02**	**2.43**	**8.66 (1.22–61.53)**	**0.03**	**2.16**
>70%	**6.35 (1.85–21.78)**	**0.003**	**2.94**	**10.93 (1.62–73.89)**	**0.01**	**2.45**
>80%	**5.25 (1.92–14.40)**	**0.001**	**3.22**	**9.32 (1.75–49.73)**	**0.009**	**2.61**
>90%	**4.15 (1.85–9.33)**	**0.001**	**3.45**	**14.55 (2.86–74.02)**	**0.001**	**3.23**

Association of SA at arbitrary threshold values of SFCC and % PMN. ^*^ Adjusted analysis included the following variables: age; sex; BMI; smoker; history of DM, CKD, liver disease, septic arthritis, and/or inflammatory arthritis; immunosuppressed; IV drug user; CRP and ESR; Charlson comorbidity index; HIV; and hepatitis.

**Table A4:** Risk factors associated with increased synovial fluid cell counts through stepwise selection procedure. Bold text indicates statistically significant p value ( <0.05 ).

SFCC						PMNs					
Variables	Coef.	95 % CI		|t|	p	Variables	Coef.	95 % CI		|t|	p
History of SA	9.67	4.06	15.30	3.41	**0.001**	History of SA	16.81	4.48	29.14	2.70	**0.008**
Inflammatory arthritis	11.11	3.30	18.92	2.82	**0.006**	ESR	0.22	0.05	0.39	2.55	**0.01**
Crystalline arthropathy	6.08	0.77	11.39	2.27	**0.03**	Crystalline arthropathy	13.93	4.48	29.14	2.55	**0.02**
						CRP	0.62	0.05	1.18	2.17	**0.03**

Stepwise forward selection procedure used to identify patient/presentation characteristics associated with SFCC and % PMNs.

**Table A5:** Sensitivity and specificity at different thresholds for septic arthritis. Bold values correlate with statistically significant ( p<0.05 ) cut-off thresholds seen in Table A3.

SFCC	Sensitivity (95 % CI)	Specificity (95 % CI)	Area under the curve (95 % CI)
> 10 000	48 % (32–65)	69 % (62–76)	0.59 (0.49–0.68)
> 15 000	42 % (26–59)	77 % (70–83)	0.59 (0.50–0.69)
>20000	**42 % (26–59)**	**82 % (76–87)**	**0.62 (0.52–0.71)**
>25000	**32 % (19–50)**	**91 % (86–95)**	**0.62 (0.53–0.70)**
>30000	**26 % (14–43)**	**95 % (91–96)**	**0.61 (0.52–0.69)**
>35000	**23 % (11–40)**	**98 % (94–99)**	**0.60 (0.53–0.68)**
>40000	**23 % (11–40)**	**99 % (96–99)**	**0.61 (0.53–0.68)**
>45000	**10 % (3–24)**	**99 % (97–99)**	**0.55 (0.49–0.60)**
% PMN	Sensitivity (95 % CI)	Specificity (95 % CI)	Area under the curve (95 % CI)
>30 %	90 % (75–97)	23 % (17–30)	0.57 (0.50–0.63)
>40 %	90 % (75–97)	25 % (19–32)	0.58 (0.52–0.64)
>50 %	90 % (75–97)	28 % (21–35)	0.60 (0.53–0.66)
>60%	**90 % (75–97)**	**33 % (26–40)**	**0.61 (0.55–0.69)**
>70%	**90 % (75–97)**	**40 % (33–47)**	**0.66 (0.59–0.72)**
>80%	**84 % (67–93)**	**49 % (42–57)**	**0.67 (0.59–0.75)**
>90%	**61 % (44–76)**	**71 % (64–77)**	**0.67 (0.57–0.76)**

Receiver operating characteristic (ROC) data for arbitrary SFCC and PMN threshold values, including sensitivity, specificity and AUC values.

## References

[bib1.bib1] Carpenter CR, Schuur JD, Everett WW, Pines JM (2011). Evidence-based diagnostics: adult septic arthritis. Acad Emerg Med.

[bib1.bib2] Coutlakis PJ, Roberts WN, Wise CM (2002). Another look at synovial fluid leukocytosis and infection. J Clin Rheumatol.

[bib1.bib3] Edwards CJ, Cooper C, Fisher D, Field M, van Staa TP, Arden NK (2007). The importance of the disease process and disease-modifying antirheumatic drug treatment in the development of septic arthritis in patients with rheumatoid arthritis. Arthritis Rheumatol.

[bib1.bib4] Ferrand J, Samad YE, Brunschweiler B, Grados F, Dehamchia-Rehailia N, Séjourne A, Schmit J-L, Gabrion A, Fardellone P, Paccou J (2016). Morbimortality in adult patients with septic arthritis: a three-year hospital-based study. BMC Infect Dis.

[bib1.bib5] Gupta MN, Sturrock RD, Field M (2003). Prospective comparative study of patients with culture proven and high suspicion of adult onset septic arthritis. Ann Rheum Dis.

[bib1.bib6] Hunter JA, Blyth TH (1999). A risk-benefit assessment of intra-articular corticosteroids in rheumatic disorders. Drug Safety.

[bib1.bib7] Jokic A, Kopcinovic LM, Culej J, Kocijan I, Bozovic M (2020). Laboratory testing of extravascular body fluids: National recommendations on behalf of the Croatian Society of Medical Biochemistry and Laboratory Medicine. Part II – Synovial fluid. Biochem Med (Zagreb).

[bib1.bib8] Kaandorp CJ, Van Schaardenburg D, Krijnen P, Habemma JD, van de Laar MA (1995). Risk factors for septic arthritis in patients with joint disease. A prospective study. Arthritis Rheumatol.

[bib1.bib9] Kennedy N, Chambers ST, Nolan I, Gallagher K, Werno A, Browne M, Stamp LM (2015). Native Joint Septic Arthritis: Epidemiology, Clinical Features, and Microbiological Causes in a New Zealand Population. J Rheumatol.

[bib1.bib10] Kinugasa M, Kobayashi D, Satsuma S, Sakata R, Shinada Y, Kuroda R (2020). The predictive value of synovial glucose level in septic arthritis. J Pediatr Orthop B.

[bib1.bib11] Li SF, Cassidy C, Chang C, Gharib S, Torres J (2007). Diagnostic utility of laboratory tests in septic arthritis. Emerg Med J.

[bib1.bib12] Lenski M, Scherer MA (2014). Analysis of synovial inflammatory markers to differ infectious from gouty arthritis. Clin Biochem.

[bib1.bib13] Long B, Koyfman A, Gottlieb M (2018). Evaluation and Management of Septic Arthritis and its Mimics in the Emergency Department. West J Emerg Med.

[bib1.bib14] Mathews CJ, Weston VC, Jones A, Field M, Coakley G (2010). Bacterial septic arthritis in adults. Lancet.

[bib1.bib15] Margaretten ME, Kohlwess J, Moore D, Bent S (2007). Does this adult patient have septic arthritis?. Jama.

[bib1.bib16] Morgan DS, Fisher D, Merianos A, Currie BJ (1996). An 18 year clinical review of septic arthritis from tropical Australia. Epidemiol Infect.

[bib1.bib17] Rasmussen L, Bell J, Kumar A, Heckman MG, Lesser E, Whalen J, Shi GG, Ledford C, Wilke B (2020). A Retrospective Review of Native Septic Arthritis in Patients: Can We Diagnose Based on Laboratory Values?. Cureus.

[bib1.bib18] Shirtliff ME, Mader JT (2002). Acute septic arthritis. Clin Microbiol Rev.

[bib1.bib19] Singh JA, Yu S (2018). Septic Arthritis in Emergency Departments in the US: A National Study of Health Care Utilization and Time Trends. Arthrit Care Res (Hoboken).

[bib1.bib20] Yu KH, Luo SF, Liou LB, Wu Y-JJ, Tsai WP, Chen JY, Ho HH (2003). Concomitant septic and gouty arthritis-an analysis of 30 cases. Rheumatology (Oxford).

[bib1.bib21] Zeller JL, Lynm C, Glass RM (2007). Septic arthritis. Jama.

